# Minimal effects of *spargel* (PGC-1) overexpression in a *Drosophila* mitochondrial disease model

**DOI:** 10.1242/bio.042135

**Published:** 2019-07-10

**Authors:** Jack George, Howard T. Jacobs

**Affiliations:** Faculty of Medicine and Health Technology, FI-33014 Tampere University, Finland

**Keywords:** Mitochondria, Mitochondrial biogenesis, Mitochondrial disease, Transcriptional co-activator

## Abstract

PGC-1α and its homologues have been proposed to act as master regulators of mitochondrial biogenesis in animals. Most relevant studies have been conducted in mammals, where interpretation is complicated by the fact that there are three partially redundant members of the gene family. In *Drosophila*, only a single PGC-1 homologue, *spargel* (*srl*), is present in the genome. Here, we analyzed the effects of *srl* overexpression on phenotype and on gene expression in *tko^25t^*, a recessive bang-sensitive mutant with a global defect in oxidative phosphorylation, resulting from a deficiency of mitochondrial protein synthesis. In contrast to previous reports, we found that substantial overexpression of *srl* throughout development had only minimal effects on the *tko^25^^t^* mutant phenotype. Copy number of mtDNA was unaltered and *srl* overexpression produced no systematic effects on a representative set of transcripts related to mitochondrial OXPHOS and other metabolic enzymes, although these were influenced by sex and genetic background. This study provides no support to the concept of Spargel as a global regulator of mitochondrial biogenesis, at least in the context of the *tko^25t^* model.

## Abstract (precis)

**Summary:** Overexpression of *spargel*, the fly PGC-1 homologue proposed as a mitochondrial biogenesis regulator, has minimal effects on the phenotype of *tko^25t^*, considered a fly model for mitochondrial disease.

## INTRODUCTION

The PGC-1 coactivators are widely considered to be global regulators of bioenergy metabolism, specifically acting to promote mitochondrial biogenesis in many different contexts (Spiegelman, 2007). However, the fact that there are three such factors encoded in mammalian genomes (PGC-1α, PGC-1β and PPRC1, also denoted as PRC) complicates their analysis, due to the combination of tissue or physiological specialization and genetic redundancy (Finck and Kelly, 2006).

In the *Drosophila* genome, a single member of the PGC-1 coactivator family, *spargel* (*srl*), is present. A *srl* hypomorph, carrying a P-element promoter insertion, was found to have decreased weight, decreased accumulation of storage nutrients in males and female sterility (Tiefenböck et al., 2010). In the mutant larval fat body there was decreased respiratory capacity and diminished expression of genes required for mitochondrial biogenesis and activity, with evidence of co-operation with the *Drosophila* NRF-2α homologue Delg, and with insulin signaling. These findings are consistent with Spargel acting as a general regulator of mitochondrial biogenesis in the fly. Many subsequent studies have been construed similarly (Mukherjee et al., 2014).

As part of a previous study of phenotypes connected with the *Drosophila* mutant *tko^25t^*, we found evidence consistent with such a role for Spargel in regard to mitochondrial functions (Chen et al., 2012). *tko^25t^* carries a point mutation in the gene encoding mitoribosomal protein S12 (Royden et al., 1987; Shah et al., 1997), which confers larval developmental delay, bang sensitivity, defective male courtship and impaired sound responsiveness (Toivonen et al., 2001). The mutant has an under-representation of mitoribosomal small subunit rRNA and is deficient in all four enzymes of the oxidative phosphorylation (OXPHOS) system that depend on mitochondrial DNA (mtDNA)-encoded subunits (Toivonen et al., 2001, 2003). The *tko^25t^* phenotype can be rescued by an additional genomic copy of the mutant *tko* locus (Kemppainen et al., 2009) and partially compensated by altered mtDNA background (Chen et al., 2012) or low-sugar diet (Kemppainen et al., 2016).

In our earlier study, flies overexpressing *srl* showed a modest but statistically significant alleviation of the mutant phenotype (Chen et al., 2012). When we later catalogued our strain collection, we concluded that this experiment may have used a strain carrying a genomic duplication of *srl* (designated *srl^GR^*, Tiefenböck et al., 2010), rather than the GAL4-dependent *srl* cDNA construct. In order to clarify the effects on *tko^25t^* phenotype of *srl* overexpression at different levels, we proceeded to combine the mutant with different *srl* constructs, having first profiled their effects on expression. In an initial experiment using *srl^GR^*, we were able to substantiate the earlier finding of a modest alleviation of developmental delay. However, this was not upheld in subsequent repeats of the experiment, nor by other strain combinations that overexpress *srl* at a higher level; nor did *srl* overexpression systematically modulate mtDNA copy number or the expression of genes for OXPHOS subunits, the mitochondrial nucleoid protein TFAM or other metabolic pathways. We thus find no consistent evidence to support a role for *srl* in boosting mitochondrial biogenesis in *tko^25t^* flies.

## RESULTS

### *srl* expression in wild-type and *tko^25t^* mutant flies

**Fig. 1.**
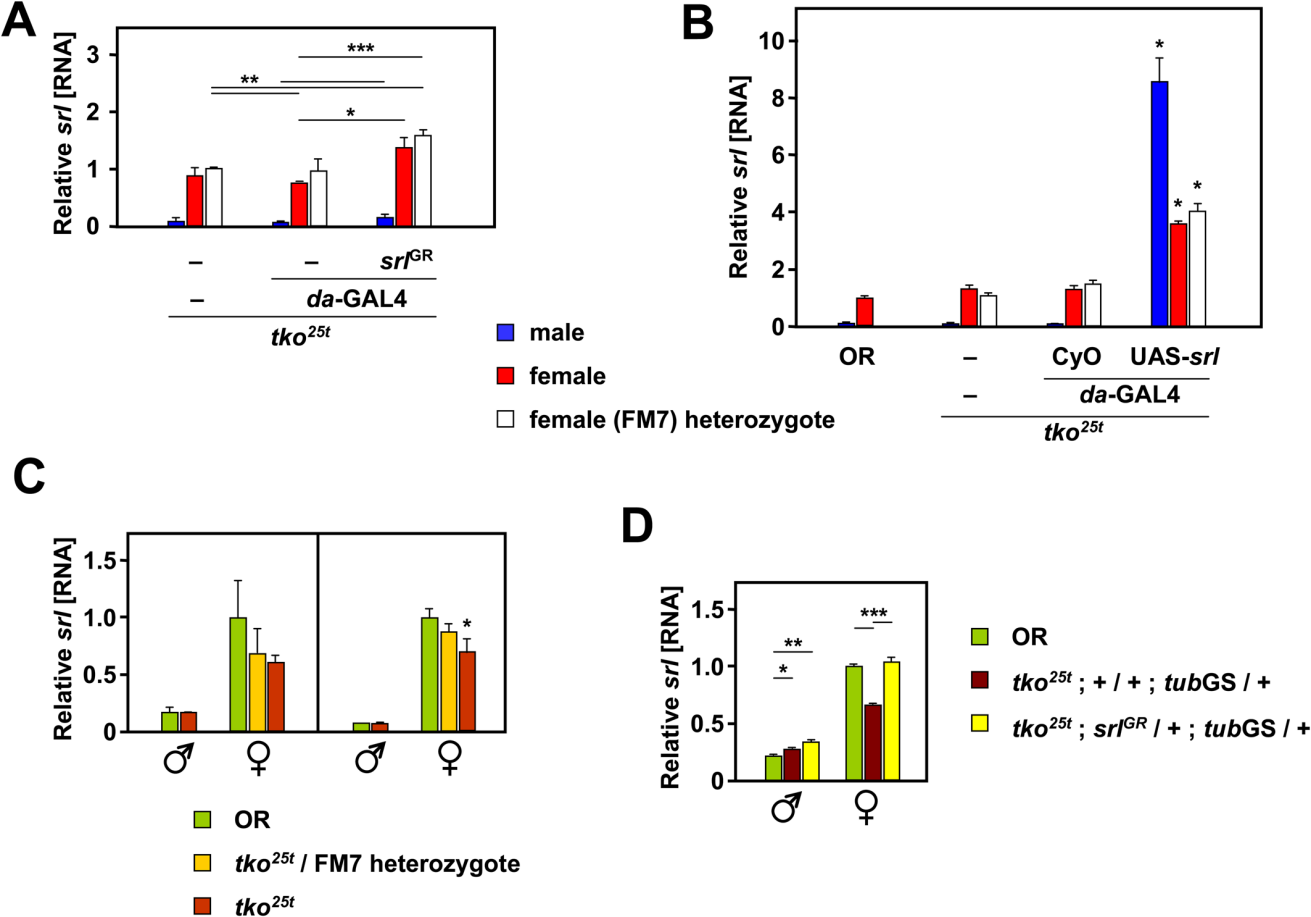
***srl* can be overexpressed by genomic duplication or using the GAL4 system.** qRT-PCR measurements of *srl* RNA in adult flies (*n*=4 batches of 10 flies) of the indicated genotypes and sex, normalized to values for control females: (A) *tko^25t^*/FM7 heterozygotes, which have a wild-type phenotype; (B–D) Oregon R (OR) wild-type. (A) Effect of genomic duplication of *srl* (hemizygosity for *srl^GR^*) with or without the additional presence of the *da*GAL4 driver in the *tko^25t^* background. (B) Effect of UAS-*srl*, with or without the *da*GAL4 driver, in the *tko^25t^* background, alongside OR. Asterisks denote significant differences between flies expressing UAS-*srl* driven by *da*GAL4 and non-expressing controls of the same sex and *tko* genotype (Student's *t*-test, *P*<0.001). (C) Effect of the *tko^25t^* background (two separate experiments separated by vertical line; repeat experiments shown to emphasize the reproducibility of the main finding, i.e. decreased *srl* expression in *tko^25t^* females compared with controls). Asterisk denotes significant difference from OR flies of the same sex (Student's *t*-test, *P*<0.05). (D) Combined effect of the *tko^25t^* background and hemizygosity for *srl^GR^*. Note that, in this experiment, we substituted the *tub*GS driver background for *da*GAL4, so as to check that the increased expression from *srl^GR^* is not due to the *da*GAL4 driver background. Asterisks denote significant differences based on Student's *t*-test with Bonferroni correction, comparing flies of a given sex between genotypes: *P<0.05, **P<0.01, ***P<0.001. Note that all statistical analyses are based on ΔC_T_ values from the qRT-PCR data, not the fold differences as plotted (see Materials and Methods).

**Fig. 2.**
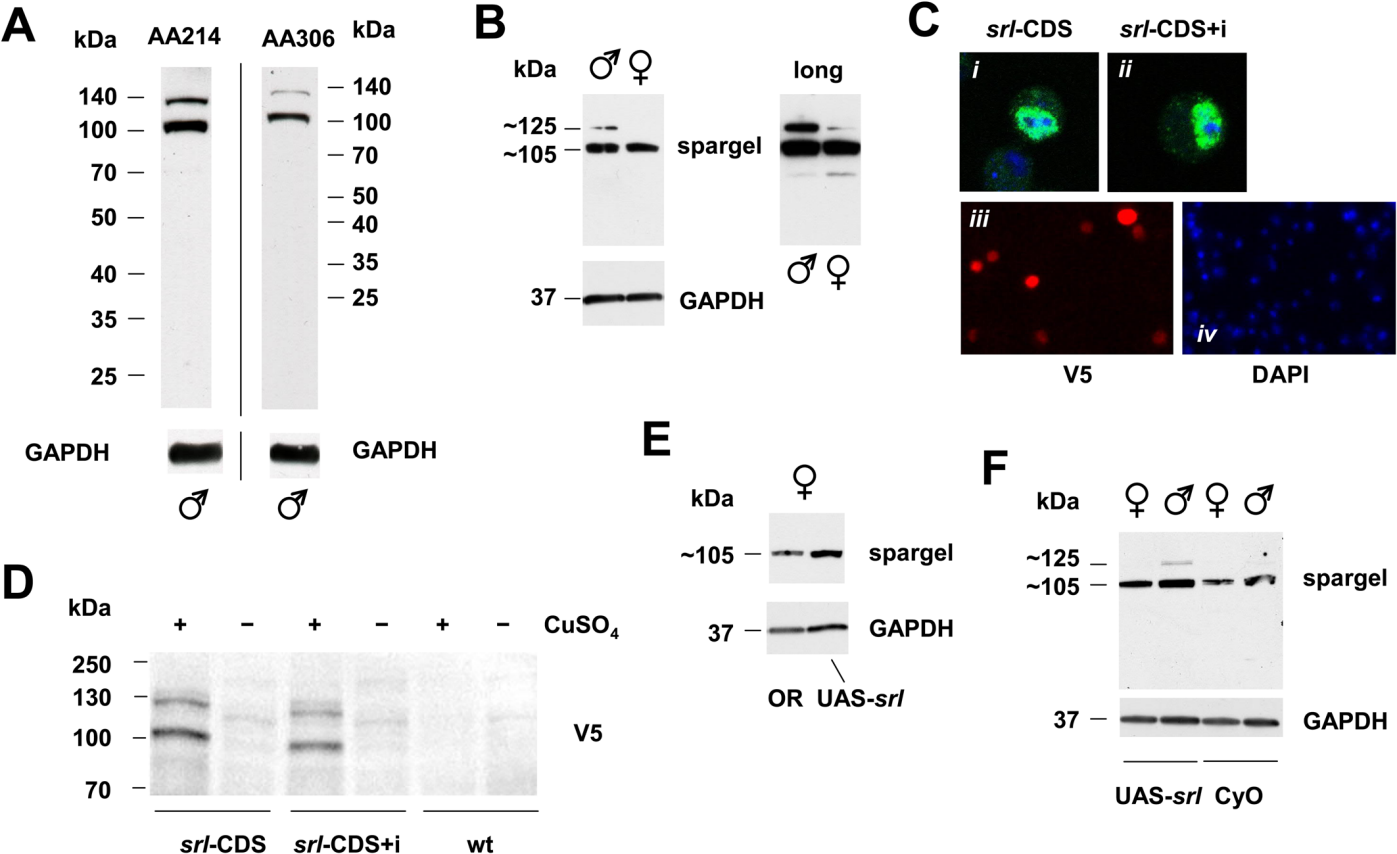
***srl* overexpression at the protein level is modest.** (A,B,E,F) Western blots of protein extracts from *Drosophila* adults of the sex and genotype indicated, probed with customized *srl*-directed antibodies AA214 (A, left-hand panel, B, E, F) or AA306 (A, right-hand panel), and with antibody against GAPDH as loading control. Sizes of molecular weight markers in kDa shown in A and D, or used to extrapolate sizes of major bands detected in B, E and F. Note longer exposure of same blot in right-hand panel of B. (C) Immunocytochemistry and (D) western blot, using V5 antibody, on cells transfected with Cu-inducible *srl*-expressing constructs (*srl*-CDS with coding sequence only, *srl*-CDS+i with intron); in C, *i* and *ii* show single cells at high magnification; *iii* and *iv* show cells probed with V5 antibody or DAPI to confirm successful transient transfection. Densitometry on Spargel ∼105 kDa band in replicate blots (*n*=3) normalized against GAPDH (mean fold-differences±s.d.) showed, for B, Spargel in males was at 1.39±0.11 times its level in females, for E, Spargel in *srl* overexpressing (UAS-*srl*/daGAL4) females was at 1.18±0.10 times its level in Oregon R wild-type females and for F, Spargel in *srl* overexpressing (UAS-*srl*/*da*GAL4) flies was at 1.53±0.01 times its level in CyO balancer/*da*GAL4 controls.

To assess the effects of *srl* overexpression in *tko^25t^* mutant flies and heterozygous controls, we first measured the extent of overexpression using qRT-PCR, after combining the relevant chromosomes carrying *srl^GR^*, UAS-*srl*, the ubiquitously acting *da*GAL4 driver, the *tko^25t^* mutation and appropriate balancer chromosomes (Fig. 1). To reproduce as closely as possible the previously studied conditions, we created *tko^25t^* flies that were hemizygous for both *srl^GR^* and *da*GAL4, even though there should be no UAS construct present (Fig. 1A). We also analyzed the sexes separately since, in initial trials, we observed a consistently higher endogenous *srl* expression in females than males. Hemizygosity for the *srl^GR^* construct conferred an increase in *srl* RNA in both sexes, proportionate to gene dosage (Fig. 1A). In contrast, UAS-*srl* driven by *da*GAL4 resulted in a more substantial increase in *srl* RNA: ∼4-fold in females and >100-fold in males (Fig. 1B). *srl* RNA was at lower abundance in *tko^25t^* females (though not males: Fig. 1C), and was restored to the wild-type level by *srl^GR^* (Fig. 1D). To test whether increased *srl* RNA due to UAS-*srl* expression was reflected at the protein level, we generated two antibodies against peptides of Spargel, which each detected a major band of approximate molecular weight ∼105 kDa and a minor band of ∼125 kDa (Fig. 2A), close to the predicted molecular weight of the protein (118 kDa). These bands were detected in both males and females (Fig. 2B: note that the ∼125 kDa band appears more faintly in females, but is always present at long exposure). The same two bands were detected in S2 cells induced to express V5 epitope-tagged Spargel after transient transfection (Fig. 2C,D). At higher magnification (Fig. 2C*i*,*ii*), immunocytochemistry revealed a ‘speckled’ nuclear localization similar to that observed by Mukherjee and Duttaroy (2013) using *srl*-GFP, providing further validation of the (AA214) antibody. UAS-*srl* driven by *da*GAL4 led to a modest increase in detected Spargel protein, based on western blot signal compared with the GAPDH loading control (Fig. 2E,F). Note, however, that this increase (∼20–50% depending on background), was proportionately far smaller than that seen at the RNA level. The large disparity in *srl* RNA between males and females (Fig. 1) was not evident in the detected protein, which was actually at a higher level in males (by ∼40%).

### *srl* overexpression has no systematic effects on *tko^25t^* phenotype

**Fig. 3.**
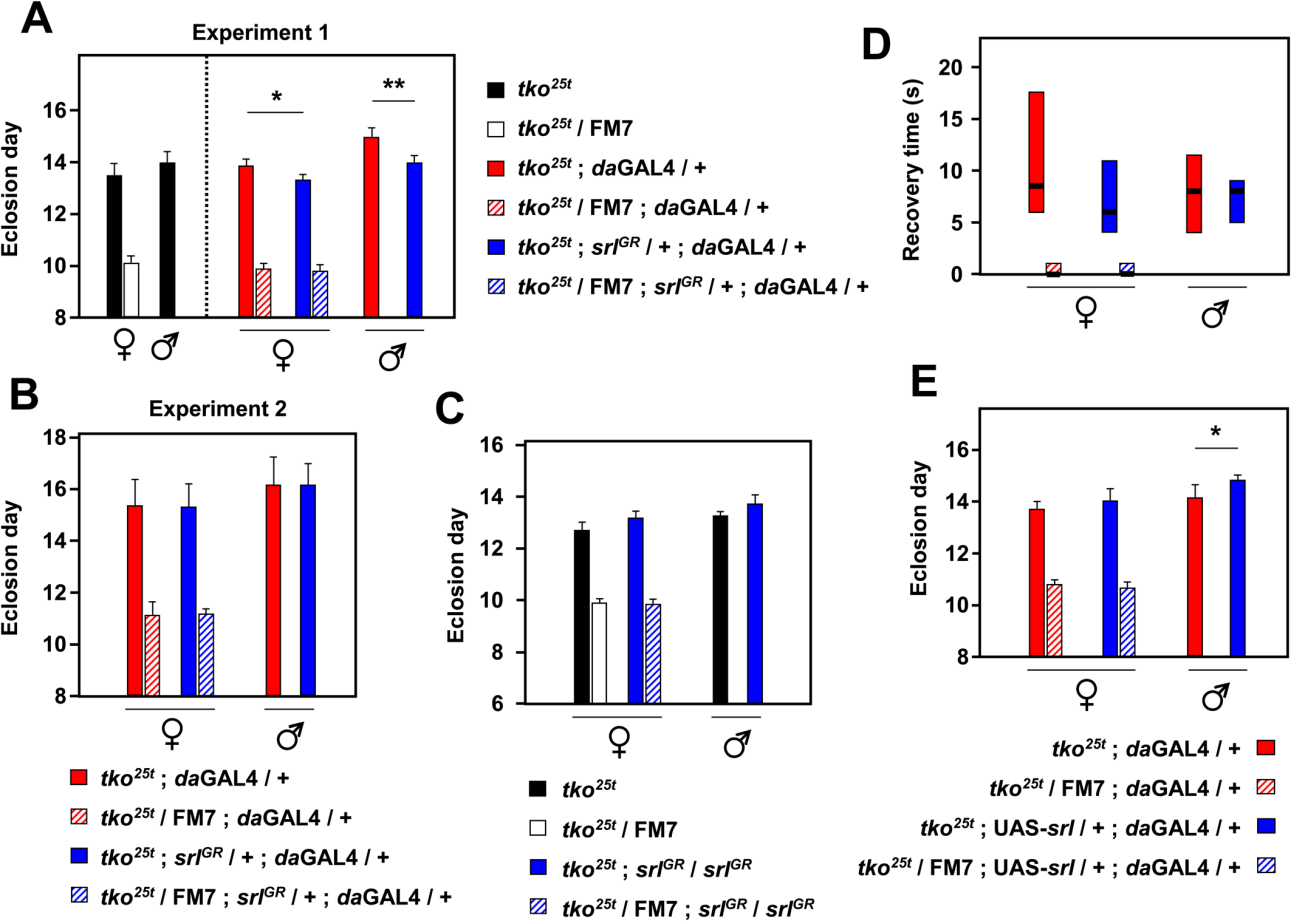
***srl* overexpression does not modify *tko^25t^* phenotype.** (A–C,E) Days to eclosion (means±s.d.) and (D) bang sensitivity (box plot, 1st to 3rd quartiles, medians as thick black bars) of flies of the indicated genotypes and sex (*n*=5 replicate vials of 10–50 flies each). Dashed vertical line in A separates the experimental and ‘*tko^25t^* only’ control (i.e. lacking both *da*GAL4 and *srl^GR^*) crosses conducted in parallel (similar controls were implemented routinely but are omitted from the other panels for clarity). A and B show two repeat experiments (denoted experiment 1 and experiment 2: a third repeat is not shown) in which *tko^25t^* ; *da*GAL4 males were crossed to *tko^25^^t^*/FM7 females either with or without *srl^GR^* as shown. The same cross was used to generate the data in D. In C, crosses for *tko^25t^* alone or in combination with homozygous *srl^GR^* were conducted in parallel, without the presence of *da*GAL4. In E, progeny carried *da*GAL4 and either UAS-*srl* or CyO from a single cross. Asterisks denote statistically significant differences between progeny flies of a given sex and *tko* genotype, either with or without the presence of an *srl* overexpression construct (Student's *t*-test; *P<0.05, **P<0.01). Two other repeats of this experiment gave similar findings.

To clarify the effects of *srl* overexpression on the phenotype of *tko^25t^* we conducted a number of tests in which we varied the overexpression construct used and the genetic background. Using the *srl^GR^* construct to produce modest overexpression we recorded a small decrease in the developmental delay of *tko^25t^* flies (Fig. 3A). However, this was influenced by the presence of the *da*GAL4 driver, since the eclosion day of *tko^25t^* flies lacking both *da*GAL4 and the *srl^GR^* construct was not significantly different from that of flies endowed with both. Furthermore, although the alleviation of developmental delay was significant in this first experiment, as inferred previously (Chen et al., 2012), it was not seen in any of the three repeats of the experiment (e.g. the one shown in Fig. 3B). There was also no significant difference in eclosion time between *tko^25t^* flies homozygous for the *srl^GR^* construct and *tko^25t^* controls in either sex (Fig. 3C). Furthermore, hemizygosity for the extra copy of *srl* produced no rescue of bang-sensitivity (Fig. 3D). More substantial overexpression of *srl* driven by *da*GAL4 using the UAS-*srl* construct did not alleviate developmental delay: rather there was a trend towards a slight deterioration, although this was significant only in one repeat of the experiment and in males only, as shown (Fig. 3E).

### *srl* expression is not altered by diet during development

Previously, Spargel was shown to mediate insulin signaling (Mukherjee and Duttaroy, 2013), which is considered the primary system linking growth to nutritional resources. In contrast, *tko^25t^* exhibits an apparently paradoxical growth retardation when cultured on high-sugar diet (Kemppainen et al., 2016), suggesting that insulin signaling has been abrogated or even reversed, for example, as a result of a counteracting signal arising from mitochondrial dysfunction. We therefore considered the hypothesis that *srl* was downregulated in *tko^25t^* by a diet-dependent mechanism and that its expression and growth-promoting function might be restored in *tko^25t^* larvae or adults cultured on minimal medium.

**Fig. 4.**
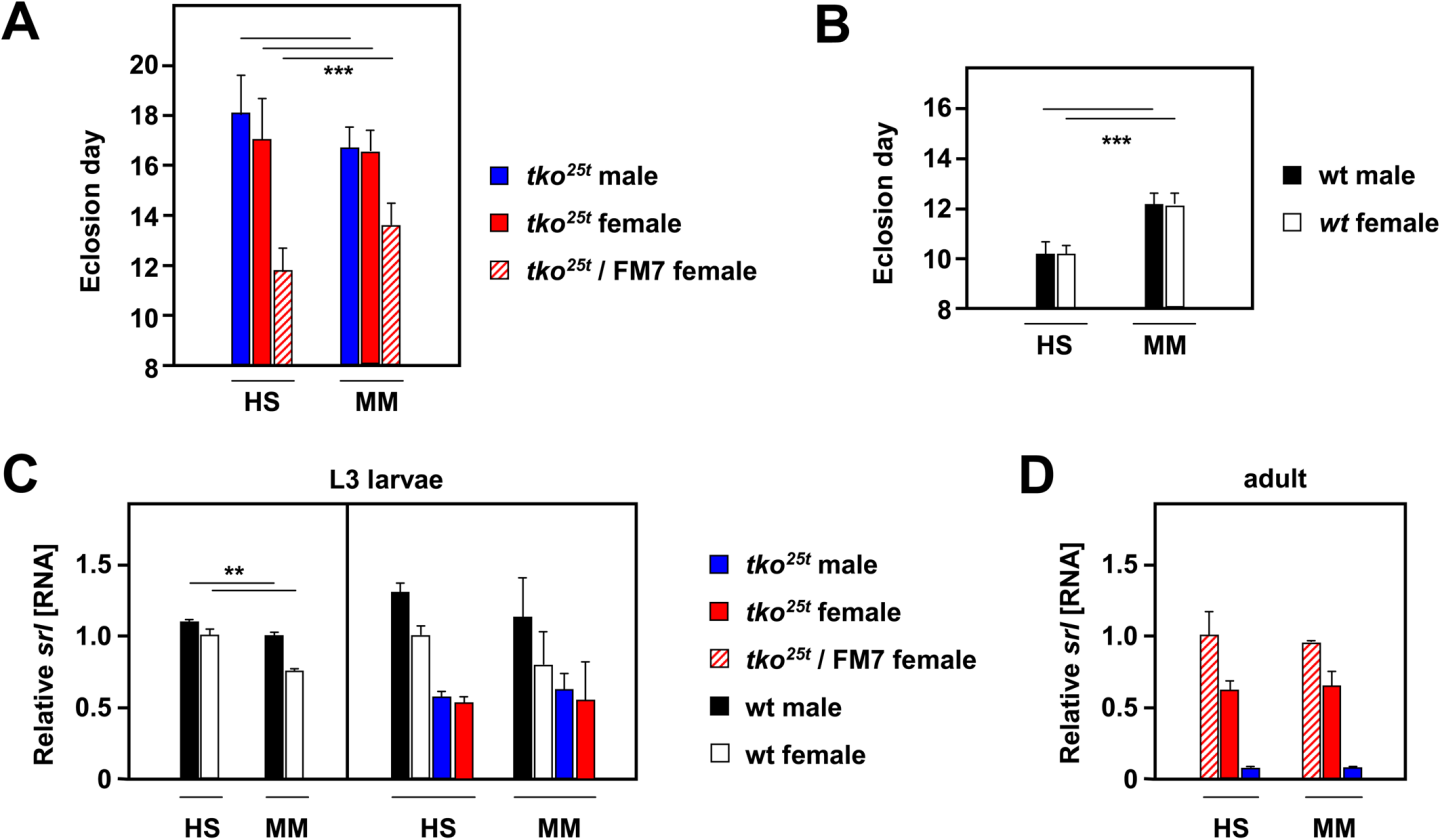
***srl* expression does not correlate with growth rate on different media.** (A,B) Means±s.d. of times to eclosion of flies of the indicated genotypes and sex on different media. HS, standard high-sugar medium; MM, minimal medium; wt, wild-type Oregon R. Flies grown in (A) bottles, *n*=739 individuals, (B) replicate vials, *n*=310 flies. (C,D) qRT-PCR measurements (*n*=4 batches of 10 flies) of *srl* RNA in L3 larvae and adult flies of the indicated genotypes and sex on the different media, normalized to values for (C) Oregon R wild-type females or (D) *tko^25^^t^*/FM7 heterozygous females. Vertical bar in C divides datasets for two separate experiments. Asterisks denote statistically significant differences between indicated classes of flies of a given sex and genotype on the different media: Student's *t*-test, **P<0.01, ***P<0.001. Note, however, that comparison of values for the equivalent classes in the experiment shown in the right-hand part of C gave no significant differences.

For this, we compared flies grown on standard high-sugar medium, containing complex dietary additives, with those grown on a minimal medium containing only agar and (10%) yeast. As previously, the low-sugar minimal medium partially accelerated the development of *tko^25t^* flies (Fig. 4A), whilst at the same time retarding that of controls (Fig. 4A,B). However, diet-induced effects on the expression of *srl* were minimal. *srl* expression in control (wild-type Oregon R) L3 larvae of both sexes was slightly decreased in minimal medium compared with high-sugar medium (Fig. 4C,D), although this was not statistically significant in all experiments (e.g. Fig. 4C, right-hand panel). *srl* expression in *tko^25t^* larvae (Fig. 4C, right-hand panel) was lower than in controls by approximately the same factor as in adults, but was unaffected by the different culture media, as was *srl* expression in *tko^25t^* adults (Fig. 4D).

### Overexpression of *srl* has no systematic effects on genes related to core mitochondrial functions

**Fig. 5.**
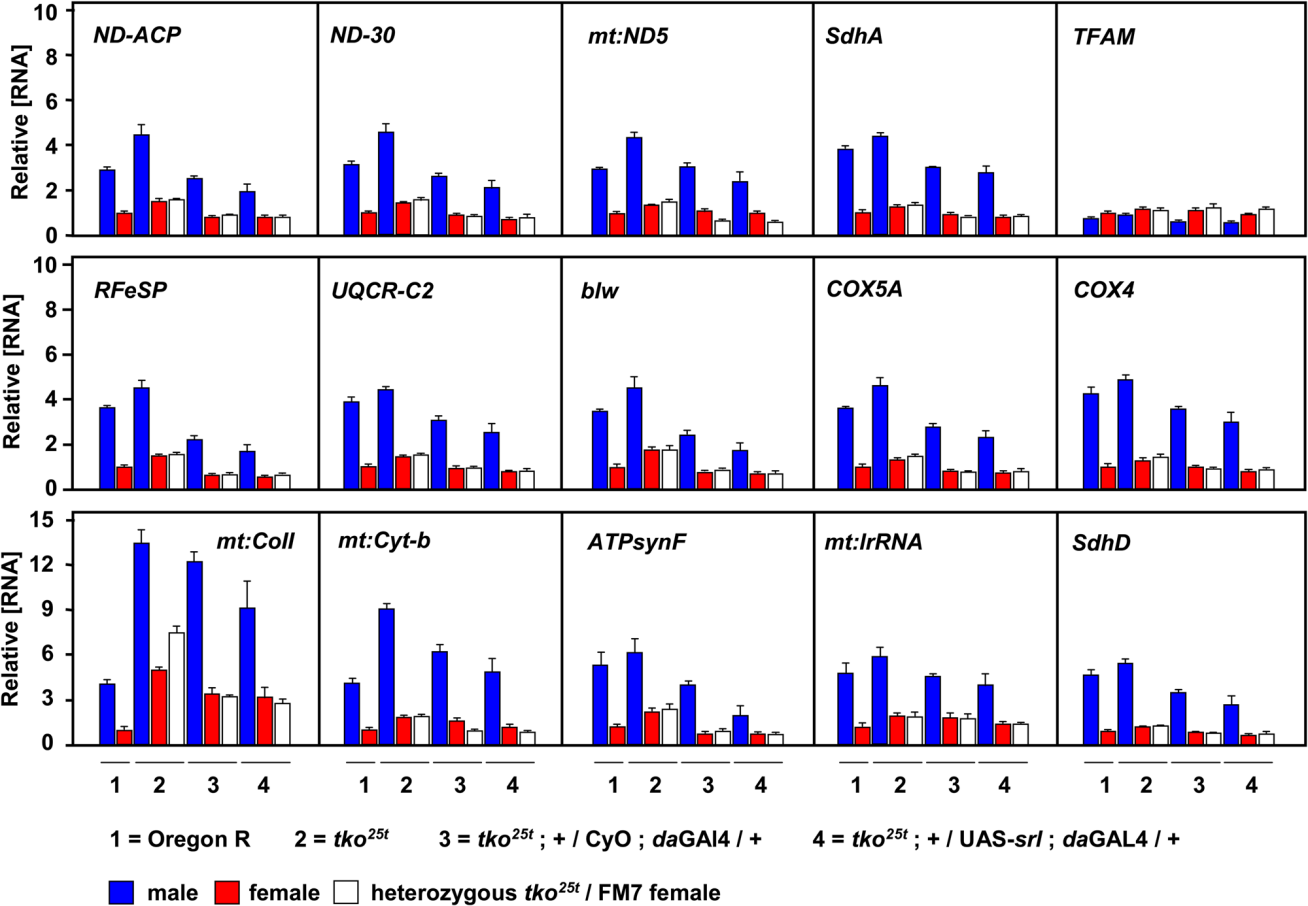
***srl* overexpression in *tko^25t^* does not increase levels of transcripts for core mitochondrial functions.** qRT-PCR measurements (*n*=4 batches of 10 flies) of RNA levels of the indicated genes (symbols as in flybase.org) in adult flies of the indicated genotypes and sex, normalized to corresponding values for Oregon R (wild-type) females. For clarity, and because expression profiles were qualitatively so similar for all genes studied (except *TFAM*), statistical estimates are omitted.

Despite the fact that *srl* overexpression had no impact on the *tko^25t^* phenotype, we explored whether such overexpression nevertheless influenced the level of mtDNA or that of transcripts related to core functions of mitochondria, specifically OXPHOS subunits and the major nucleoid protein TFAM (Fig. 5). With the exception of TFAM, all genes studied showed a similar profile of expression in the different strains tested, with higher relative expression in males, higher expression in the *tko^25t^* background, including *tko^25t^* heterozygotes over the FM7 balancer, attenuation of this increase by the *da*GAL4 driver and further slight attenuation by UAS-*srl*. These observations are consistent with expression levels being determined by sex and by genetic background, possibly involving effects on the *RpL32* reference transcript, rather than by *srl* expression, which followed a different pattern (Fig. 1B). They provide no support for any enhancing effect of *srl*. In the case of TFAM, expression was slightly lower in males than in females, and was little affected by *da*GAL4 or UAS-*srl* (Fig. 5, top right). Note that *srl* overexpression was verified (Fig. 1B) in the same RNA samples.

**Fig. 6.**
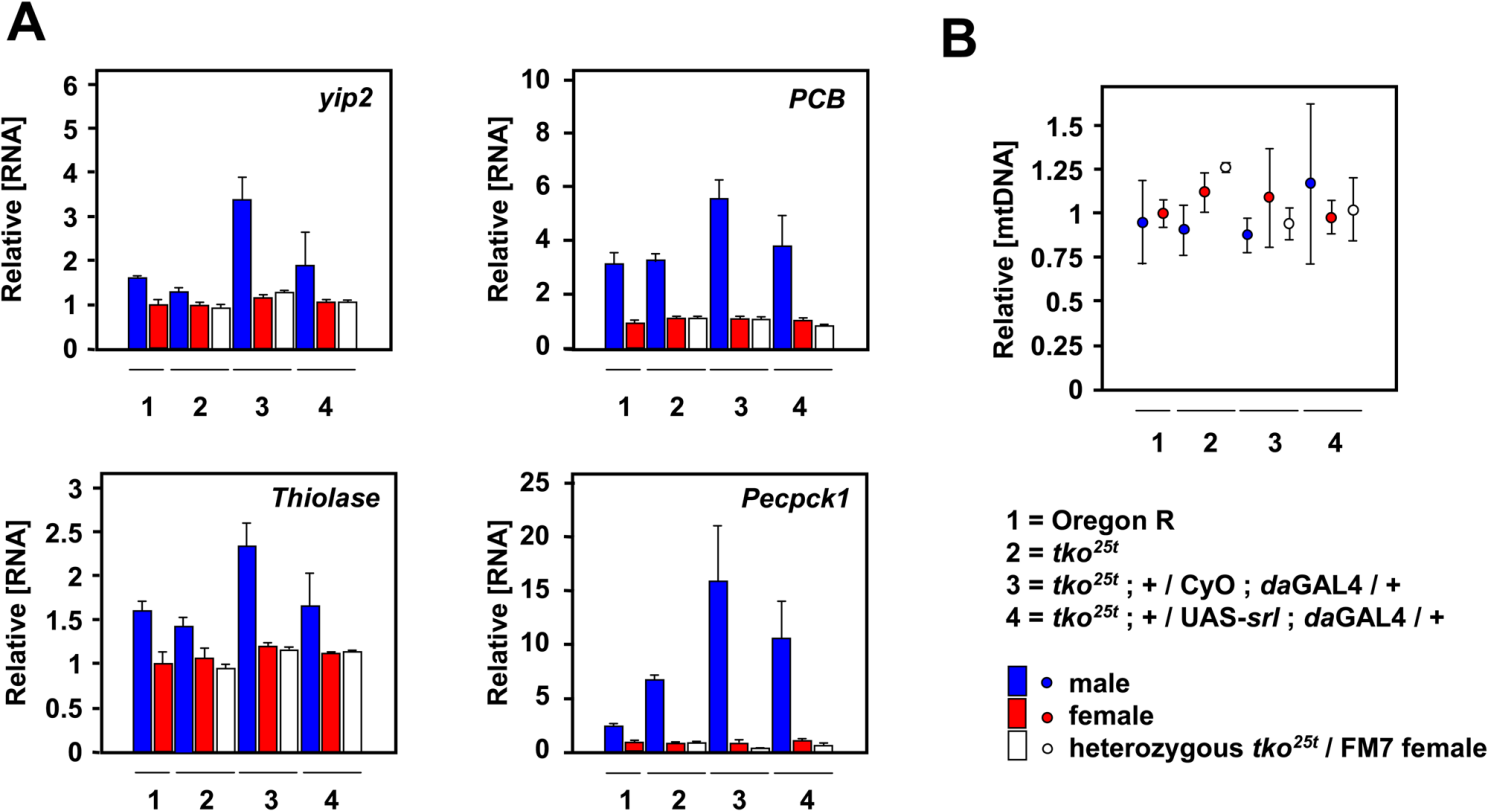
***srl* overexpression in *tko^25t^* does not increase mtDNA copy number or transcript levels for other metabolic enzymes.** (A) qRT-PCR measurements of RNA levels (*n*=4 batches of 10 flies) of the indicated genes (symbols as in flybase.org) in adult flies of the indicated genotypes and sex normalized to corresponding values for Oregon R (wild-type) females. Statistical analysis confirmed a significant effect of both sex and genotype and of interaction between these factors for all four genes (two-way ANOVA, *P*<0.001). However, as for the genes studied in Fig. 5, narrowly comparing expression of flies of a given sex in the presence of *da*GAL4, with or without UAS-*srl*, showed no significant differences (Student's *t*-test with Bonferroni correction), despite the general trend of slight decrease in males. (B) qPCR measurements of mtDNA copy number (*n*=4 batches of five flies), means±s.d. normalized to Oregon R females. There were no significant differences between groups (one-way ANOVA).

In previous studies, the expression of genes for some of the enzymes participating in other metabolic pathways known to be influenced by PGC-1 homologues in various contexts, such as lipid catabolism, including beta-oxidation of fatty acids (Huang et al., 2017), or gluconeogenesis (Rhee et al., 2003), were found to be upregulated in *tko^25t^*, both in larvae (Kemppainen et al., 2016) and adults (Fernández-Ayala et al., 2010). We therefore tested whether *srl* over-expression driven by *da*GAL4 was able to influence the expression of genes for such enzymes in *tko^25t^*, despite the absence of any effect on growth rate. Once again, using the same materials as in the experiment shown in Fig. 5, we found no significant effect of *srl* overexpression on the transcripts of two genes for enzymes of fatty acid oxidation (*yip2* and *Thiolase*) and two for gluconeogenesis (*PCB* and *Pepck1*), although all of them were affected by sex, by genetic background and by the interaction of these factors (two-way ANOVA, Fig. 6A).

Next, we measured relative mtDNA copy number in *tko^25t^* and control flies, with and without *srl* overexpression. ANOVA revealed no significant differences between groups (Fig. 6B). Thus, *srl* overexpression does not appear to influence mitochondrial or metabolic functions in *tko^25t^* in any systematic way.

## DISCUSSION

Previous studies, where the expression of *srl* was downregulated either in the whole fly or in a specific tissue, suggested a global role for *srl* in growth regulation. Here we tested whether overexpression of *srl* was able to compensate the phenotype of *tko^25t^*, a mutant with decreased mitochondrial biosynthesis and which grows slowly. We found that *srl* RNA was at decreased levels in *tko^25t^* flies (Figs 1 and 4) even when cultured in minimal medium where the growth defect is partially alleviated. As suggested in a previous study, we initially detected a small compensatory effect of *srl* overexpression in *tko^25t^* flies endowed with an additional copy of *srl* (Fig. 3A). However, further repeats (e.g. Fig. 3B) and trials with *srl^GR^* in two copies (Fig. 3C) failed to substantiate any rescue of developmental delay or bang-sensitivity (Fig. 3D). Even the much more substantial *srl* overexpression produced by UAS-*srl* (Fig. 1B) was ineffective (Fig. 3E). Overexpression of *srl* had no effect on mtDNA copy number, nor on transcripts of genes connected with mitochondrial OXPHOS or other metabolic pathways. There are several potential explanations for these essentially negative results that we now consider, noting that that *srl* overexpression was also previously found not to compensate for decreased OXPHOS capacity resulting from a mutation in the adenine nucleotide translocase (Vartiainen et al., 2014).

### Translational and post-translational regulation

The first possibility is that, as suggested by the lack of congruence between RNA and protein levels, *srl* is translationally regulated, negating any effect of overexpression. Translational regulation is well established (see recent reviews by Zhao et al., 2019; Shi and Barna, 2015), applies to mitochondrial biogenesis (Zhang and Xu, 2016), and is prominent in early development (Winata and Korzh, 2018; Barckmann and Simonelig, 2013), playing roles in axial specification and other processes in *Drosophila* (Wilhelm and Smibert, 2005; Kugler and Lasko, 2009). It is also a cardinal feature of the integrated stress response (Ryoo and Vasudevan, 2017), which can be activated by mitochondrial dysfunction.

A second idea is that *srl* might be post-translationally regulated, which could also override effects of overexpression. Post-translational regulation is brought about by many different mechanisms (Gill, 2004; Lee et al., 2005; Johnson, 2009; Bauer et al., 2015; Narita et al., 2019; Klein et al., 2018). Many of them have been documented as affecting the PGC-1 family in mammals (reviewed by Austin and St-Pierre, 2012), which is also subject to differential splicing (Meirhaeghe et al., 2003; Martínez-Redondo et al., 2015). The two antibodies that we generated against Spargel detect the same bands on western blots, validated by epitope tagging in S2 cells. The higher molecular weight band (∼125 kDa) probably represents the predicted full-length protein of 119 kDa. The nature of the processing that generates the major (∼105 kDa) band is unknown, but can be considered a suggestive indicator of post-translational regulation of Spargel.

### *tko^25t^* signaling

A third possible explanation for the finding that *srl* overexpression fails to modify the *tko^25t^* growth phenotype could be that the mutation might elicit a growth-inhibitory signal, overriding any effect of *srl*. Therefore, we should not just dismiss the conventional view that the PGC-1 coactivators are global regulators of mitochondrial biogenesis. Such a function may apply in many other physiological contexts. Indeed, if Spargel acts in this way as a ‘master switch’, its effects may still be masked by metabolic signaling at a lower level in the hierarchy of gene regulation. Based on previous data (Kemppainen et al., 2016), a strong candidate for growth regulation in *tko^25t^* is ribosomal protein S6 kinase (S6K), which is influenced by multiple signaling pathways including mTOR (Magnuson et al., 2012), insulin/Akt (Manning, 2004) and AMPK (Mihaylova and Shaw, 2011). Contradicting this idea, Mukherjee and Duttaroy (2013) found that *srl* can partially override defects in cell growth mediated by defective insulin/mTOR signaling and that mutants in S6K can be rescued by *srl* overexpression. However, since S6K regulation in *tko^25t^* seems to be at the level of the protein itself, not its phosphorylation, *srl* over-expression may be insufficient to negate it.

### A different role for *spargel*

Spargel may also play a broader role than just promoting mitochondrial biogenesis. Although mitochondrial biogenesis is reciprocally affected by PGC-1α knockout (Lin et al., 2004; Leone et al., 2005) and overexpression (Lehman et al., 2000; Lin et al., 2002), the PGC-1 family also impacts thermogenesis in brown fat (Uldry et al., 2006), neuromuscular differentiation (Lin et al., 2002; Handschin et al., 2007), hepatic gluconeogenesis (Yoon et al., 2001) and oxygen radical detoxification (St-Pierre et al., 2006). As a coactivator, PGC-1 interacts with sequence-specific transcription factors which specify the genes to be regulated, but the known transcriptional targets of *srl* are not limited to those involved in mitochondrial biogenesis (Tiefenböck et al., 2010), and it has elsewhere been implicated in various cell differentiation and cell survival programs, or in functional maintenance during aging (Tinkerhess et al., 2012;Wagner et al., 2015; Merzetti and Staveley, 2015; Diop et al., 2015; Ng et al., 2017; Staats et al., 2018). Rera et al. (2011) reported an increase in mitochondrial markers in flies globally overexpressing *srl*. However, this is also consistent with a general enhancement of muscle differentiation. Finally, we should not exclude the possibility that *srl* could promote mitochondrial biogenesis by an effect other than on transcription, even if this would not affect the levels of mtDNA/TFAM, mitoribosomes or mitochondrial mRNAs in *tko^25t^* flies, nor modify the *tko^25t^* phenotype. However, since there is no precedent for a transcriptional coactivator influencing the levels of target proteins but not their mRNAs, this must be considered highly unlikely.

### Issues in fly genetics

Our initial results using *srl^GR^* (Fig. 3A) were consistent with previous studies, but partial rescue of *tko^25t^* could not subsequently be reproduced. The reasons for the discrepancy are not clear, but we posit that both the original, apparent rescue and its non-reproducibility are most probably attributable to genetic background effects, and subject to genetic drift during stock maintenance. Unknown and therefore uncontrolled environmental variables may also have played a role. Note, in addition, that our initial finding (Fig. 3A) indicates a small negative effect of the *da*GAL4 driver. Transgenes, drivers and deleterious mutations are routinely maintained over balancer chromosomes. Balancers are preferable to homozygosity, so as to prevent the inadvertent selection of suppressors, and are unavoidable in cases where homozygosity is lethal. However, balancers also allow new mutations to accumulate, protected from negative selection. These too potentially compromise the reproducibility of effects on mild phenotypes, such as developmental delay in *tko^25t^*. Although genetic drift can be minimized by alternating rounds of homozygosity and rebalancing, in practice it cannot be completely prevented. Whilst burdensome, our study highlights the value of multiple repeat experiments to confirm quantitatively minor phenotypic variations, preferably with retesting in different backgrounds. Such measures are nevertheless much easier to implement and interpret in *Drosophila*, compared with mammalian models where inconsistent or strain-dependent findings abound.

Although we found no effects on phenotype, mtDNA copy number or gene expression from *srl* overexpression in *tko^25t^*, it should be noted that all our assays were conducted on whole adult flies. Therefore, our findings largely reflect the situation in the muscle-rich thorax, where mtDNA and its transcription and translation products are at their most abundant (Calleja et al., 1993). Although we cannot exclude an *srl*-dependent effect in some tissue other than muscle, to detect it would require extensive dissection procedures or the use of highly tissue-specific drivers. The present results provide no basis upon which to embark on such a study.

## MATERIALS AND METHODS

### *Drosophila* strains and culture

The *srl^GR^* and UAS-*srl* strains (Tiefenböck et al., 2010), both supplied over a CyO balancer, were a kind gift from Christian Frei (ETH Zürich, Switzerland). The *tko^25t^* strain, originally sourced through Kevin O'Dell (University of Glasgow, UK), was backcrossed into Oregon R background (Toivonen et al., 2001) and maintained long-term in our laboratory over the FM7 balancer. The Oregon R wild-type and *da*GAL4 driver strains were originally obtained from Bloomington Stock Center and the *tub*GS driver was the kind gift of Scott Pletcher (University of Michigan, USA). All stocks were maintained at room temperature and grown experimentally in plugged plastic vials at 25°C on a 12 h light/dark cycle in standard high-sugar medium (HS, Kemppainen et al., 2016) or, where specified in figures, in a minimal medium (MM) consisting of agar, 10% yeast and standard antimicrobial agents (0.1% nipagin and 0.5% propionic acid, Sigma-Aldrich).

### Molecular cloning

Genomic DNA was extracted from adult *Drosophila* and used as a PCR template with chimeric gene-specific primers to amplify *srl* from the start codon up until, but not including, the stop codon. The chimeric primers contained EcoRI and NotI restriction sites for restriction digestion and insertion into the copper-inducible plasmid pMT-V5/HisB (Thermo Fisher Scientific), resulting in the introduction of an in-frame C-terminal V5 epitope tag. A primer deletion strategy was used on this plasmid to create an intronless version of *srl* tagged with V5. Both resulting plasmids were sequence-verified before use in transfections.

### Developmental time and bang-sensitivity assays

Three replicate crosses were set up and tipped five times to fresh vials for egg laying, as previously described (Kemppainen et al., 2009). The mean developmental time to eclosion (at 25°C), as well as bang-sensitivity, were measured as described previously (Kemppainen et al., 2009). Unweighted means and standard deviations of eclosion day for each sex and inferred genotype were then computed for each cross, and used in statistical analyses, generally applying Student's *t*-test (unpaired, two-tailed) to compare the mean eclosion day of flies of a given sex and genotype with and without the expression of a given *srl* overexpression construct. For bang-sensitivity, medians and quartiles of recovery time for flies of a given sex and genotype were plotted in a box-plot format.

### RNA analysis

Total RNA was extracted from batches of ten 2 day-old flies and from L3 (wandering stage) larvae using a homogenizing pestle and trizol reagent as previously described (Kemppainen et al., 2016). cDNA was synthesized using the High-capacity cDNA Reverse Transcription Kit (Thermo Fisher Scientific) according to manufacturer's instructions. Expression levels were determined by qRT-PCR using Applied Biosystems StepOnePlus™ Real-Time PCR System with Fast SYBR™ Green Master Mix kit (Applied Biosystems) with, as template, 2 μl of cDNA product diluted 10-fold, in a 20 μl reaction, together with 500 nM of each gene-specific primer pair as follows (all given 5′ to 3′, gene symbols in the following list following current practice in flybase.org): *RpL32* (CG7939), TGTGCACCAGGAACTTCTTGAA and AGGCCCAAGATCGTGAAGAA; *ND-ACP* (CG9160), ACAAGATCGATCCCAGCAAG and ATGTCGGCAGGTTTAAGCAG: *ND-30* (CG12079), AAGGCGGATAAGCCCACT and GCAATAAGCACCTCCAGCTC; *mt:ND5* (CG34083), GGGTGAGATGGTTTAGGACTTG and AAGCTACATCCCCAATTCGAT; *SdhA* (CG17246), CATGTACGACACGGTCAAGG and CCTTGCCGAACTTCAGACTC; *TFAM* (CG4217), AACCGCTGACTCCCTACTTTC and CGACGGTGGTAATCTGGGG; *RFeSp* (CG7361), GGGCAAGTCGGTTACTTTCA and GCAGTAGTAGCCACCCCAGT; *UQCR-C2* (CG4169), GAGGAACGCGCCATTGAG and ACGTAGTGCAGCAGGCTCTC; *Blw* (CG3612), GACTGGTAAGACCGCTCTGG and GGCCAAGTACTGCAGAGGAG; *COX5A* (CG14724), AGGAGTTCGACAAGCGCTAC and ATAGAGGGTGGCCTTTTGGT; *COX4* (CG10664), TCTTCGTGTACGATGAGCTG and GGTTGATTTCCAGGTCGATG; *mt:CoII* (CG34069), AAAGTTGACGGTACACCTGGA and TGATTAGCTCCACAGATTTC; *mt:Cyt-b* (CG34090), GAAAATTCCGAGGGATTCAA and AACTGGTCGAGCTCCAATTC; *ATPsynF* (CG4692), CTACGGCAAAGCCGATGT and CGCTTTGGGAACACGTACT; *mt:lrRNA* (CR34094), ACCTGGCTTACACCGGTTTG and GGGTGTAGCCGTTCAAATTT; *SdhD* (CG10219), GTTGCAATGCCGCAAATCT and GCCACCAGGGTGGAGTAG; *srl* (CG9809), GGAGGAAGACGTGCCTTCTG and TACATTCGGTGCTGGTGCTT; *yip2* (CG4600), GTCCTCCTCCACCGATGGTAT and CAAAGCCGGTTGATTCCAAGG; *Thiolase* (CG4581), GGAGTCCGCACACCCTTTC and TGCAGCAATGACAAAAGCGAG; *PCB* (CG1516), AATCGGTGGCGGTCTACTC and TTGCCCACTATGTACGACTCG; *Pepck1* (CG17725), TGATCCCGAACGCACCATC and CTCAGGGCGAAGCACTTCTT. Mean values were normalized first against *RpL32* and then against an arbitrary standard, namely wild-type (Oregon R) adult females, except where stated. Primer pairs were routinely validated based on uniform melting profiles, and standard curves showing efficiencies of at least 90%. For further details of qRT-PCR methods in our laboratory see Fernandez-Ayala et al. (2009) and Saari et al. (2019).

### mtDNA copy number measurement

Batches of five adult flies of a given sex were crushed in 500 µl DNA lysis buffer (75 mM NaCl, 50 mM EDTA, 20 mM HEPES/NaOH, pH 7.8). 5 µl of 20% SDS and 20 µl of Proteinase K (10 mg/ml, Thermo Fisher Scientific) were added to each sample and vortexed to mix. Samples were briefly centrifuged, left on a heat block at 50°C for 4 h, then vortexed and centrifuged at 16,000 ***g****_max_* for 1 min to pellet debris. Supernatants were decanted and nucleic acid was precipitated with 420 µl of isopropanol with repeated inversion and overnight incubation at −20°C. Samples were centrifuged at 16,000 ***g****_max_* for 30 min at 4°C to pellet the DNA, which was washed with 500 µl of ice-cold 70% ethanol. Final pellets were left to air dry for 10 min, then resuspended in 100 µl of TE buffer (10 mM Tris/HCl, 1 mM EDTA, pH 7.8) overnight at 50°C. DNA concentration was measured by nano-drop spectrophotometry and samples were diluted to 2.5 ng/µl. Relative DNA levels of *RpL32* (single-copy nDNA) and *mt:lrRNA* (16S, mtDNA) were determined by qPCR using Applied Biosystems StepOnePlus™ Real-Time PCR System with Fast SYBR™ Green Master Mix kit (Applied Biosystems), using as template 2 μl of DNA in a 20 μl reaction, together with gene-specific primer pairs each at 500 nM, as follows (all shown 5′ to 3′): *RpL32*–TGTGCACCAGGAACTTCTTGAA and AGGCCCAAGATCGTGAAGAA; *mt:lrRNA*–ACCTGGCTTACACCGGTTTG and GGGTGTAGCCGTTCAAATTT. mtDNA copy number was inferred from the cycle-time difference (ΔC_T_) of the two test genes, i.e. 2expΔC_T_. Standard deviation (s.d.) was calculated from the mtDNA copy number values in a genotype group, and means (and s.d. values) were finally normalized to those of Oregon R females.

### Protein analysis

Batches of ten 2-day-old adult flies were crushed in 100 µl of lysis buffer (0.3% SDS in PBS plus one EDTA-free cOmplete™ Protease Inhibitor Cocktail Tablet, Roche), incubated for 15 min and centrifuged at 15,000 ***g****_max_* for 10 min (all manipulations at room temperature). Supernatants were decanted and protein concentrations determined by the Bradford assay. Aliquots of 50 μg protein in SDS-PAGE sample buffer containing 0.2 M dithiothreitol were heat-denatured for 5 min at 95°C then electrophoresed on AnyKD midi criterion™ gels (Bio-Rad) in ProSieve™ EX running buffer (Lonza). Transfer to Nitrocellulose membrane (Perkin-Elmer) was performed using ProSieve™ EX transfer buffer (Lonza). Membranes were blocked in 5% non-fat milk in PBS-0.05% Tween (Medicago) for 30 min at room temperature, with gentle agitation. Primary antibody diluted in the same buffer was added and reacted at 4°C overnight. After three 10 min washes, secondary antibody was added in the same buffer containing 5% non-fat milk for a further 2 h. Membranes were washed twice for 10 min in PBS-0.05% Tween and then for a final 10 min in PBS. Primary antibodies and dilutions were as follows: Srl214AA (against peptide CFDLADFITKDDFAENL) and Srl306AA (against peptide CPAKMGQTPDELRYVDNVKA), custom rabbit polyclonal antibodies (21st Century Biochemicals, both 1:5000), GAPDH (Everest Biotech EB06377, goat polyclonal, 1:5000), anti-V5 (Thermo Fisher Scientific, mouse monoclonal #R96025, 1:10,000). Appropriate HRP-conjugated secondary antibodies (Vector Laboratories, 1:5000). 5 ml of Luminata™ Crescendo Western HRP substrate solution (Merck) was added for 5 min before imaging with a Bio-Rad imager.

### Transfections and immunocytochemistry

Transfection and induction of S2 cells with V5-tagged *srl* constructs and subsequent staining for imaging was performed as previously (González de Cózar et al., 2019). The primary antibody used was mouse anti-V5 (Life Technologies) along with the corresponding Alexa Fluor^®^ 488 or Alexa Fluor^®^ 647 secondary antibodies (Abcam), with image acquisition by confocal microscopy.

### Image processing

Images have been cropped and/or rotated for clarity and optimized for contrast and brightness, but without other manipulations.

### Statistical analysis

Data were analyzed using Student's *t*-test (two-tailed, with Bonferroni multiple-test comparison where indicated), one-way or two-way ANOVA, as appropriate (Microsoft Excel and GraphPad Prism). *n* numbers (batches of flies, representing at least 20 individual flies in total in each case, or replicate vials, representing 50–250 individual flies in total in each case) as indicated in figure legends. No exclusion criteria were applied. Note that, for statistical analysis of quantitative PCR data, ΔC_T_ values were used, because they are normally distributed, whereas the extrapolated fold-changes are not, having been subjected to an exponential transformation. Thus, to apply standard statistical tests such as ANOVA or Student's *t*-test, the ΔC_T_ values must be used.
